# Sharing of Needles and Syringes among Men Who Inject Drugs: HIV Risk in Northwest Bangladesh

**DOI:** 10.1371/journal.pone.0148276

**Published:** 2016-02-05

**Authors:** M. Kamal Pasa, Kazi Robiul Alom, Zubaida Bashri, Sten H. Vermund

**Affiliations:** 1 Department of Anthropology, University of Rajshahi, Rajshani, Bangladesh; 2 Institute of Environmental Science, University of Rajshahi, Rajshani, Bangladesh; 3 Vanderbilt Institute for Global Health and Department of Pediatrics, Vanderbilt University School of Medicine, Nashville, Tennessee, United States of America; University of Athens, Medical School, GREECE

## Abstract

**Introduction:**

Injection drug use is prevalent in northwestern Bangladesh. We sought to explore the context of needle/syringe sharing among persons who inject drugs (PWID), examining risk exposures to blood-borne infections like the human immunodeficiency virus (HIV) and hepatitis in a region where these dual epidemics are likely to expand.

**Methods:**

We used a qualitative research approach to learn about injection practices, conducting 60 in-depth interviews among PWID. We then conducted 12 focus group discussions (FGDs) that generated a checklist of salient issues, and followed up with personal observations of typical days at the drug-use venues. Content and interpretative frameworks were used to analyze qualitative information and socio-demographic information, using SPSS software.

**Results:**

We found that needle/syringe-sharing behaviours were integrated into the overall social and cultural lives of drug users. Sharing behaviours were an central component of PWID social organization. Sharing was perceived as an inherent element within reciprocal relationships, and sharing was tied to beliefs about drug effects, economic adversity, and harassment due to their drug user status. Carrying used needles/syringes to drug-use venues was deemed essential since user-unfriendly needle-syringe distribution schedules of harm reduction programmes made it difficult to access clean needles/syringes in off-hours. PWID had low self-esteem. Unequal power relationships were reported between the field workers of harm reduction programmes and PWID. Field workers expressed anti-PWID bias and judgmental attitudes, and also had had misconceptions about HIV and hepatitis transmission. PWID were especially disturbed that no assistance was forthcoming from risk reduction programme staff when drug users manifested withdrawal symptoms.

**Conclusion:**

Interventions must take social context into account when scaling up programmes in diverse settings. The social organization of PWID include values that foster needle-syringe sharing. Utilization and impact of risk reduction programmes might be improved with expanded clean needle/syringe distribution at times and venues convenient for PWID, better trained and non-judgmental staff, and medical assistance for health problems, including drug withdrawal symptoms.

## Introduction

The risk to persons who inject drugs (PWID) of needle/syringe sharing (NSS) behaviour is most notable for transmission of human immunodeficiency virus (HIV) and hepatitis C virus (HCV), though other infections are also transmitted via NSS, including hepatitis B virus, human T-lymphotropic viruses, and malaria. In Bangladesh, PWID are a key vulnerable population, given that 9^th^ round national serological surveillance found PWID to have nation’s highest rate of HIV (5.3%) and HCV (>50%). Injection of drugs is especially prevalent in the northwest, near India [1]. Several studies have found a high frequency of NSS frequency among PWID in Bangladesh [1–4]. Three-quarters of PWID in Dhaka either borrowed or lent needles/syringes during their last injection event; the average size of the sharing network for PWID who shared during their last injection varied from 1.0 to 2.8 [3]. However, the context of sharing of needles and syringes among PWID is not well understood in northwest Bangladesh where injection drug use (IDU) is highly prevalent. While prior studies have described the frequency of NSS[1–4] and the prevalence of HIV and HCV [1], the social basis of sharing has received scant attention in Bangladesh. The minimum which has been demonstrated in the studies across societies and cultures lacking generalizability, holistic interpretation of cause(s), and creates controversies as to embrace with competing paradigm and methodological choices. NSS has been found to be a ritualized social practice and a conscious act of collegial reciprocity [5, 6] and is considered normative practice to many respondents in one study [2]. Such sharing both forges and renews interpersonal bonds and also builds mutual trust among PWID [5, 6]. In turn, peer pressure and relationships encourage sharing [7, 8]. However, others have argued that NSS is motivated by the perception that sharing maximizes drug effects, and may have little to do with rituals or cementing social relationships [9–13]. Thus, the social determinants of NSS have been variably reported across studies.

The economics of sharing hinge upon unemployment [14, 15] and general economic disadvantage [7, 14–16] that discourage spending on sterile injection equipment. Politico-administrative systems also pose a threat to persons carrying needles/syringes [8, 16], and further discourage the sale of sterile needles/syringes to drug users [12, 16–19]. Harm reduction programme often provides inadequate coverage for clean needle/syringe distribution [16]. Sterile needles/syringes are not provided in prisons, though drugs are often provided by guards or visitors with impunity [16]. Physical and Psychological issues are exacerbated by homelessness [14], depression and anxiety [20], and drug withdrawal symptoms that may supersede other health concerns [7, 16]. Finally, the physical setting for drug injection is often a “shooting gallery” where NSS is endemic [7, 21]. We hypothesize that NSS behaviours are steeped in social, economic, politico-administrative, psychological, and physical context.

In Bangladesh, studies report high NSS frequencies among PWID, but its causes remain largely unexplored [22–29]. Predictors of NSS may vary depending upon with whom a PWID intends to share, shortages of sterile supplies, perceptions of the importance of germ-free needles/syringes, and other subjective views [3]. One Bangladeshi study reports NSS to be attractive to PWID because sharing is cheap (10%), convenient (18%), and considered as is the norm for IDU (15%) [2]. Many PWID believe that NSS with family members or persons who appear healthy is unlikely to transmit germs. PWID also commonly believe that germs will be washed out of a needle/syringe either by water[27] or blowing into the needle and wiping off residual moisture with a cloth. While such observations offer insights into diverse proximate social and cultural contexts of NSS, detailed assessments of risk behaviours and barriers to uptake of risk reduction strategies are needed [30]. Multiple factors influence risk-taking within a specific societal and cultural context [31].

We have used intensive interviewing and detailed ethnographic research to address these contextual questions [32]. First, we explored characterization of NSS of the PWID in northwest Bangladesh. Second, we probed the social and cultural situations under which PWID are motivated to share needles-syringes. Our goal has been to provide a more holistic understanding of the context of NSS to help design better risk reduction programmes and further inform public health policy.

## Methods

We recruited PWID who injected at least once in the last month in Rajshahi city. Over six months of field work from August 2011-January 2012. We recruited 140 eligible respondents in different drug-using venues through snowball sampling techniques. Among them, 60 respondents were selected purposively for in-depth qualitative interviews. These included active injectors, PWID who switched from non-injection heroin use to Injection and vice versa, married and unmarried PWID, PWID who were homeless and those living with families, and PWID who had NSS experiences within the past six months. Before starting data collection, the interviewing researcher (KP) built personal rapport with prospective participants to encourage them to feel confident sharing key life events around drug use.

After clarifying to the prospective participant the purpose of the research, the key issues to be addressed, and informing them of confidentiality measures, we obtained written informed consent. For illiterate persons and PWID who did not wish to put their signatures or thumb impression on the consent form, we obtained witnessed verbal informed consent. Consent confirmed by a signature or thumbprint was provided by 120 respondents, while 20 others gave their verbal consents.

For socio-demographic information, we administered a survey based on a close ended questionnaire to the 140 respondents in different drug-using venues and other locations convenient to the respondents. The questionnaire was supported with interview guidelines to clarify what specific terms used in a given question meant. Internal validity was built into the questionnaire by cross-checking answer with other related questions.

For qualitative information, we used multiple data collection tools, including in-depth interview, FGD, and standardized observations. Sessions were conducted in such a way that respondents felt empowered to answer questions in a non-judgmental atmosphere; interviewers were trained to avoid value-laden expressions. Following respondents’ answering an open ended questionnaire, we conducted the 60 in-depth interviews that generated a number of compelling case studies. We started sessions only when respondents had no symptoms of heroin withdrawal, a positive temperament towards the interview, and agreement to discuss their lives with us for about an hour. We encouraged open ended engagement; if PWID wished to discuss issues apart from our script, we accepted this fully. We also changed interview venues, when requested, to avoid risk of disclosure and interference from strangers or known persons. PWID were permitted time breaks as needed and could also discuss issues with other PWID who might be nearby, if they wished. Interviews lasted no more than one hour.

Twelve FGD were conducted, guided by a pre-planned checklist with probing cues to go into details on specific issues. Each session included 9–11 participants. We employed different indepth interview participants to assist organizing the FGD session smoothly. Those participants were on board in different FGD sessions, none was allowed to be a participants of FGD session in two times. The session lasted 60–90 minutes. PWID anticipating imminent withdrawal symptoms were excused from participation. We sought to engage all FGD participants in providing their opinions, drawing out the less responsive participants. During the field work period, personal observations by trained study personnel as to lifestyles and life events of PWID were made at drug-use venues, with real-time documentation.

We continued our in-depth interviews until we found saturation in the issues being discussed. However, the FGD were conducted based on prior planning and were all conducted as originally designed. To check the validity of the information, we did a triangulation of our approaches, examining themes that emerged from the survey, the in-depth interviews, and the FGD, examining how answers to the same question emerged from the three types of engagements. We cross checked themes derived from multiple respondents.

All but three of the in-depth interviews were recorded. In lieu of recordings for the three in-depth interviewees who preferred not to be recorded, we took detailed field notes. Immediately after returning from the field each day, we completed a transcript from the recordings and/or the field notes. Given the legal complexities of drug use and drug dealing in Bangladesh, only de-identified data were used with respondents’ names and drug using venues indicated by unique numeric codes and alphabetic codes respectively during the transcription process. Our analytic approach did not require us to re-identify data. The subjects’ right to privacy was maintained throughout. Confidentiality was also maintained by safeguarding entrusted information (a locked cabinet in a locked room). During the research process, no information was shared except within the research team. After submission of the report to Bangladesh University Grants Commission (UGC), the data became within the UGC purview as the sole authority to disclosure of information as per their rules and regulations for studies which they fund. Finally, we preserved all the raw data, hard copies in a locked closet and computerized data in a locked folder in the study computer. The UGC maintains standard security measures for safeguarding the report.

Qualitative information was analyzed using content and interpretative frameworks, using Health Belief model constructs. In brief, interview transcripts produced from recording and field notes were analyzed in several steps. Step one was the organizing of the data. We read and re-read all the interview transcripts until we had a general understanding of the data, summarizing it in writing. We identified and differentiated relevant responses between and across the questions and topics. In Step two, we sought out the emerging themes by identifying and organizing ideas and concepts through close examination of words/phrases used frequently, meaning of the language used by participants, unexpected responses, and life stories. We then categorized and coded the key ideas and concepts. Identifying and labelling the themes into codes depended upon specific content in relation to the given research theme being addressed. We did not have prior hypotheses prior to going through the content of the interview transcripts. The Step three consisted of collapsing different categories within principal overarching themes, when justified. We sought possible and plausible explanations for these overarching themes through interpreting their meaning and significance, examining similarities and dissimilarities of topic-specific statements.

Survey data were analyzed in terms of frequency distribution and percentage, average and interquartile range with SPSS® version 20 software (IBM, Armonk, New York, USA). Qualitative data were analyzed without the use of research software.

The University Grants Commission of Bangladesh (UGC; http://www.ugc.gov.bd/, accessed September 3, 2015) supported the grant, which was vetted by an array of research UGC review committees prior to and post data collection. One panel was responsible for approving study methods and another provided the ethical review and approval. The final report was accepted by the UGC after a comprehensive review. We accommodated UGC panel feedback at several stages of the research.

## Results and Discussion

### Socio-demographic profiles

All subjects were male. We found evidence of women who injected drugs, but they were hidden, unreachable through snowball sampling presumably due to the conservative Bangladeshi sociocultural context. The age range (Table 1) was 16 to 48 years (median = 26.5, interquartile range [IQR] 23–29). Respondents’ literacy levels were very low; nearly two-thirds of PWID were illiterate (Table 1). Nearly three of four PWID were married, about half lived alone, and about half lived in a slum or on the street (floating/homeless; Table 1). Occupations were diverse, with many who were unemployed or were underemployed in very low-paying positions (Table 1). While unemployed men, thieves, smugglers, drug dealers, and beggars represented 61.4% (86/140) of PWID, the others had occupations of wide diversity. At the income extremes, day laborers and rickshaw pullers had low salaries, while contractors and bus drivers had higher salaries. This diversity was demonstrated in the personal and family income data, though very few persons had truly middle class incomes (Table 1).

**Table 1:** Socio-demographic profile for 140 persons who inject drugs in Rajshani, Bangladesh.

Characteristics	Number (%)
**Age in years**	
16–19	13 (9.3%)
20–24	37 (26.4%)
25–29	60 (42.9%)
30–34	17(12.1%)
35–48	13 (9.3%)
**Educational background**	
Illiterate	89 (63.6%)
1–5 Class	24 (17.1%)
6–10 Class	18 (12.9%)
≥11 Class	9 (6.4%)
**Marital status**	
Currently married	95 (67.9%)
Unmarried	37 (26.4%)
Divorced or Separated	8 (5.7%)
**Occupation**	
Unemployed	19 (13.6%)
Rickshaw or rickshaw-van puller	15 (10.7%)
Contractor or Bus driver	11 (7.9%)
Student	11 (7.9%)
Illicit drug seller	17 (12.1%)
Clinic or diagnostic middleman or Technician	8 (5.8%)
Rag picker	11 (7.9%)
Thief or smuggler	32 (22.9%)
Beggar	7 (5.0%)
Transport helper, Welder, Day laborer	9 (6.4%)
**Monthly personal income in taka and (US $)**	
<3000 (<$4.21)	7 (5.0%)
350–525 ($4.21–6.33)	11 (7.9%)
526–875 ($6.34–10.54)	84 (60.0%)
876–1050 ($10.55–12.65)	18 (12.9%)
>1050 (>$12.65)	20 (14.3%)
**Monthly family income in taka and (US $)**	
<3000 (<$36.14)	17 (12.1%)
3000–4000 ($36.14–48.19)	26 (18.6%)
4001–6000 ($48.20–72.29)	61 (43.6%)
6001–8000 ($72.30–96.39)	25 (17.8%)
>8000 (>$96.39)	11 (7.9%)
**Living arrangements**	
With wife and children	32 (22.9%)
With friends or others	52 (3.5%)
With relatives	7 (5.0%)
**Type of dwelling**	
Floating/Homeless	36 (25.7%)
Slum living alone	31 (22.1%)
Own house	64 (45.7%)
Relative's house or other	9 (6.5%)
**Total**	**140 (100%)**

83 taka per U.S.$1.00, at the time of the study.

### Injection drug-using behaviours

PWID typically perceived taking drugs as a bad habit with adverse health and social consequences.

*“Drugs gradually destroy our lives*, *addiction makes our bodies so vulnerable that how long we would be alive*, *only God knows*.*” (Comment made*, *in various ways*, *in nearly all key informant interviews and FGD with PWID)*

PWID reported diverse health problems, especially since they had begun using drugs. These included: weight loss, appetite loss, drowsiness, insomnia, headache, breathing problems, nausea, jaundice, sexually transmitted infections (STI), and many other uncharacterized symptoms. PWID were faced with physical violence, including torture by family members and outsiders, stigma, marginalization, social exclusion at the societal level, and psychological abuse, e.g., bad comments, abasement, and other verbal abuse. PWID reported difficulties in maintaining recognized social norms, values, and expectations. To meet the costs of drugs, many PWID engaged odd jobs, both legal and illegal. These included small scale stealing both in and out of their homes, trafficking of goods or drugs, sale of household utensils, sale of blood, and menial work that had low social standing. PWID also reported that their families were often victimized through poor treatment by society.

*“None can avoid withdrawal symptoms*. *To meet the drug requirement at that time*, *anybody can do anything*.*” (FGD with PWID)*.

To avoid drug withdrawal symptoms, PWID often thought of how to stop using drugs, but they reported failing in almost all cases. Users reported that their bodies did not function well without the use of drugs. PWID reported being unable to avoid frequent drug withdrawal symptoms, including cramping and convulsions of muscles, severe headaches, and debilitating nausea. PWID reported social pressures against drug users that led to anxiety and depression, that in turn motivated more drug use. Since PWID passed most of their time with other PWID, in a friendly environment for IDU, they were also influenced to continue use.

*“It is such a bad thing*, *but users hardly avoid drug use once they become regular users*.*” (In a number of key informant interviews with PWID)*

Specific drugs (the name used by users is listed first) that were used included heroin, *tedijecik/Tidigesic®* (oral buprenorphine, also injected) and psychotropic drugs that were typically injected, such as *ejeium/Sedil*®/diazepam, phenergan, *Avil*®/pheniramine, *Obsenil®*/clomipramine, *Largactil®*/chlorpromazine, either in cocktail form or separately. Amongst, buprenorphine was most popular and is being increasingly combined with other psychotropic drugs. In the local vernacular, this is called *Coktail*. The cocktail form was perceived to enhance drug effects such that the more expensive buprenorphine could be used at lower doses, minimizing the costs of drugs to achieve the same effects. Heroin users who did not typically inject often reported switching to injection when there was a heroin shortage in local market, typically due one of the frequent raids by Narcotics and Police forces. This crisis also equally prevailed for illicit buprenorphine, though psychotropic drugs were quite available in the local pharmacies.

*“Injection is the only alternative to [non-injected] heroin*. *Several times I have to switch from heroin to injection due to shortage of heroin*. *Compared to other drugs*, *unavailability of heroin is very common in the local market and its cost also fluctuate very often*, *sometime go beyond my purchasing capacity*. *And so*, *I gradually turn to injecting drugs*.*” (key informant interview with PWID*, *23 year old rickshaw puller)*

Preferred injection sites in the body were variable, including veins and/or muscles on the hands, arms, legs, and around the genitals. Persons shifting from non-IDU to IDU typically started by injecting into their muscles, then moving to venous injection to get a stronger drug effect. When muscles were hard from repeated injections and veins became blocked, uses shifted to injecting elsewhere on their bodies.

PWID most often took drugs in a group, sharing costs of drugs and needles/syringes. We found an overwhelmingly tendency to share injected drugs. Non-sharing with separate up-draw of the drug from the small ampule was considered clumsy and difficult.

*“Whenever we decide to take drugs*, *even when we are in a period of drug withdrawal*, *we look forward to have more partners to join in each particular shooting*. *This costs less and brings high feelings*.*” (FGD with PWID who were participants in a harm reduction programme)*.

Nearly all subjects, both new and old users, had experienced NSS in their drug-using lifetimes. Users reported that sharing made the shooting event more enjoyable with enhanced reciprocal bonding, i.e., helping each other in times of need, and allowing sharing of life events. Few PWID expressed many concerns over NSS. One measure was reported to enhance a sense of safety and/or to enhance drugs flowing more smoothly while injecting. Before using a used needle-syringe, coagulated retained blood was wiped off with paper or cotton. Even the harm reduction programme participants believed that this would enhance safety, despite having gotten information on health risks associated with NSS.

*We usually clean needles and syringes with paper*, *cotton*, *cloth that is worn*, *mouth blow*, *and water–whatever is around us*. *This not only enhances smooth drug flow but also keep us safe from any kinds of germ*. *(FGD with PWID who were participants in a harm reduction programme)*

The number of NSS partners ranged from three to five at a typical shooting event, but partner numbers could fluctuate depending on several factors. Sharing among partners was organized either *ad hoc* among those PWID present at that time (some of whom might be having drug withdrawal symptoms) or via a prior arrangement with known peers. Physical presence at a drug-using venue in a particular period sometimes was impeded by the cost of drugs, family pressure of not allowing a PWID to go outside, work pressures, raids by police and/or community residents, or a lack of fear of drug withdrawal symptoms at a particular time.

Needle-syringe sharing partners were largely known to each other and good mutual understandings were reported. Needle-syringe -sharing peers shared a common “occupation”, namely IDU, and they often congregated for much of a given day. At times, hitherto unknown PWID could become drug and needle-sharing partners, since addicts moved frequently from one spot to another during the frequent times of drug shortage. Due to law enforcement pressures, the drug market was very unstable and crises of periodic drug shortages were reported. In such crisis circumstances, users felt obliged to offer some portion of their drugs to persons close to them, both if they were present during drug use or if they arrived during the times of preparation or injection. Not to offer drugs and needles/syringes would be a threat to the overall relationship, since when the user would have their own urgent needs in the future, the given contact could be expected to respond in kind, sharing available drugs.

*“We are friends in need*. *We often share drugs and food with each other at the spot when someone have a financial crisis*. *We could not avoid them*, *similarly they could not avoid us as well*. *We continue our friendship because of our own interest (FGD with PWID)*.

Thus the drug-NSS network operates pragmatically, avoiding as many withdrawal events as possible.

### Contexts of Needle-Syringe Sharing

Diverse interconnected social and cultural contexts motivated the PWID towards NSS. We present seven constructs in which this was presented by participants, summarizing common responses and themes within the interviews and FGDs.

#### (1) Sharing partners as fictive kin

PWID conceived their sharing partners to be forms of kin and family. Isolated from their biological or marriage families and marginalized from the society as a whole, PWID built up distinct social organizations to facilitate their drug-using lifestyles.

*“Friends are everything to me*. *You [non-users] have all-family*, *parents*, *and work*. *But for me what is left*. *Because of my drug habit*, *I have no place at my family and society as well*. *I am treated like waste to them*. *So the friends are my family now*. *(key informant interview with PWID*, *a rag picker)*

Participants reported that their social organizations were based on respect and love for one another. Like family, they shared food, non-food items, and each other’s joyous and sorrowful moments, i.e., their life events. Membership to the social network was only open to persons who injected drugs and non-injecting heroin users. Members of the group were obliged to support each other in addressing their problems. During a typical day, when someone joined the group in a particular venue, he would be greeted in his own local language. The social group boundary was maintained by avoiding non-drug users and by concealing group activities. However, PWID reported interacting in a friendly manner with other marginalized persons, such as street-based commercial sex workers, rag pickers, and homeless persons. Taking drugs alone, i.e., outside a social network, was seen as an inappropriately individualistic attitude, impractical, and was depreciated.

#### (2) Drug-needle-syringe sharing group to aid survival

Staying in a group was perceived to enhance survival as a drug addicts.

“*Suppose I am going to steal something to manage drug cost*. *The support of a reliable one is very important at that time*. *He could alert me whether someone is coming or not to the spot*. *Otherwise*, *there would be the possibility of my being caught red-handed*. *(key informant interview with PWID*, *occasional thief and smuggler)*

Collective action was required to manage the cost of drugs, needles, and syringes, and to cope with adverse social situations. Odd-jobs, including illegal ones, were reported as the occupations of a majority of PWID (Table 1). Weekly incomes of the typical PWID were at subsistence levels, between 701–875 taka (US$8.45–10.54; Table 1). PWID could only rarely get good jobs, impeded by addict identities and poor educational levels. Illegal work included stealing, goods smuggling across borders and drug dealing. These “jobs” were aided by joining or forming a group of like-minded confederates. Furthermore, staying in a group helped addicts manage stigma, harassment, and violence, most prevalent when a drug addict was alone. Staying in a group served as a non-verbal message about their unity to potential abusers or harassers. When necessary, mutual assistance and resistance could be secured by the group.

#### (3) Heightened drug experiences

PWID commonly believed that taking drugs in a group facilitated heightened drug effects, making drug use more enjoyable than when taken alone. While taking the drug, conversation about the drug and its effect stimulated users intensely. In addition, continuation of conversation on various unmet needs or issues could be shared.

*“We get real pleasure of drug while taking it in a group*. *Taking drug alone could not satisfy our thrust*.*” (FGD with PWID*, *harm reduction programme participant)*

#### (4) Coping with poverty

Poor economic conditions affected drug and NSS, since any given individual might not have enough money to buy drugs for himself. Convincing others to share in the drug purchase mitigated any one person’s inability to purchase drugs. Ensuring equal distribution of drugs also motivated PWID towards NSS. After buying the drug through cost sharing, PWID needed to distribute the drug in some accord with the given financial contributions. Because of the typically small size of a drug-containing ampule, equitable distribution was possible through the syringe’s volume markings. After pushing the distributed amount in one’s body, the syringe was given to another and so on. Cost minimization attitudes discouraged PWID from spending money on sterile needles/syringes.

*Very often we do not have enough money to buy the drug ampule*. *So*, *we try to accumulate money around us to buy it*. *Then*, *we push out equally according to one’s contribution*. *(FGD with PWID)*

Hence, PWID preferred to reuse needles/syringes and save all their resources for drugs and food.

#### (5) Harassment when buying needles and syringes

A substantial risk of abuse and harassment was reported by our participants when they sought to buy needles/syringes from pharmacies, including risks of carrying the needles/syringes to venues for needle exchange.

*“Usually we might not stay on the spot for a long after taking the drug*. *After taking the drugs*, *we either give the needle and syringe to another or throw it out at the spot*. *Some of us keep the used one in an isolated place covering with paper or dead leaves or polythene [sic] for using the next time or for needle-syringe exchange*. *Carrying needles or syringes generates a potential risk of disclosure and harassment by police*, *the local power block*, *and family also*.*” (FGD with PWID)*

*“Most often I could not able to find my old used needles and syringes in the place I have kept it*. *Sometimes*, *I have argument with another as he exchanged my used needle-syringe as his possession to the harm reduction worker to get the new one*.*” (key informant interview with PWID*, *a Technician)*

Accessing new needles/syringes from harm reduction programmes was not at all a smooth transaction. Against the legal provisions set forward by the Bangladesh Narcotic Department, harm reduction programmes only provided new needles/syringes to PWID who brought the old, used one. The Narcotic and Security officials see the offering of new needles/syringes to be a kind of promotion of drug abuse, whereas the staff of the risk reduction programmes considered harm reduction to occur by removing used needles/syringes from the community, reducing social and cost barriers to buying new ones. The workers see their encouragement of using new needles/syringes as realistic since even though drug abuses are not able to stop their use immediately, at least they can reduce threats to transmit infections to others.

Carrying used needles/syringes posed substantial risks of harassment from police and local power brokers. Risk reduction programme people were perceived by PWID as having failed to advocate adequately as to the essence of needle/syringe exchange to police, narcotics dealers, and local power blocs. Moreover, there were no secure storage places to store old needles/syringes at drug using venues. Any such item typically was expropriated by other users, and arguments would ensue about returning used needles/syringes to harm reduction workers. Furthermore, cross mobility across spots and time of day induced by the practicalities of drug user’s life were unavoidable condition. The limited and fixed or inconvenient schedules to drug users of the harm reduction workers often resulted in missed opportunities for needle/syringe exchanges as harm reduction field workers were guided by the non-flexible programme design. Participants also highlighted the unequal relationships between harm reduction workers and the addicts. Field workers could be prejudiced, judgmental, and contemptuous in their attitudes. Feuds or favoritism might influence the fair distribution of needles, syringes, and condoms. Harm reduction workers sometimes treated addicts as dirty, criminals, liars or otherwise bad persons, with consequent mistreatment and discrimination in needle/syringe and condom distribution. Moreover, harm reduction workers’ forceful admonitions to quit using drugs would discourage some PWID from staying in contact with the workers. The harm reduction workers’ attitudes lie in their beliefs about drug addiction, echoing popular social images that IDU is a heinous and socially harmful act. Training as to how staff should respectfully treat the addicts in the field might not adequately changing preconceived notions about addicts. PWID reported that staff would behave badly, seeing this as justified to get PWID out of addiction. Staff considered their work to be humanistic, like a Messiah on behalf of the drug users.

#### (6) Misconceptions about HIV/HCV risk

PWID believed that sexual intercourse was the only mode of transmission of HIV, and that this came mainly from infected commercial sex workers.

*We do not encounter any STIs after injecting drugs for a long time*. *Why would the injecting practices cause STIs*? *This is happened only to those who have sex with sex workers*. *(FGD with PWID)*

Even participants of harm reduction programmes had misconceptions about such fundamental topics as HIV transmission. Misconceptions were many: breathing in presence of an HIV-infected person could transmit HIV; washing through “mouth blow” or water would make a used needle/syringe germ-free; jerking or booting (drawing up blood before injection) made the injection germ-free; healthy, good looking, and/or familiar persons are not infected with STIs such as HIV. Respondents stated that HIV-infected persons would always have bodily signs and symptoms of the infection.

Those misconceptions had a social grounding, as these shared views maintained a distinct sub-culture different from mainstream. Either staying with family or alone, they were being treated as ‘other’ excluded from social events and family decision-making. Their otherness enhanced group formation among drug-using peers with a distinct social organization maintaining shared understandings and practices. PWID had diverse misconceptions relating to HIV and HCV, including that both were rare diseases. Their constructed ideas of HIV/HCV were based on their real-life experiences and learning via the subculture’s societal views. (Mainstream, non-drug-using persons also shared these diverse misconceptions about HIV and HCV transmission.) Within a group of PWID, the seniors, long term experienced drug users, were valued for their imparted knowledge. These senior PWID felt an obligation to make newcomers aware of key concepts within the injecting community. Persons who used drugs for a long time and did not have apparent infections were used as models for the harmlessness of NSS when minor cleanliness measures were promulgated. Those ideas was reinforced among group members through normal chatting and frequent ritualized sharing of needle-syringe.

#### (7) Low self-esteem

Given the stigma associated with drug addiction, PWID were marginalized in society; depression and anxiety were very common. Due to mistreatment even from persons nearest to them, PWID gradually lost their self-confidence and did not believe that they could do anything good for themselves, their families, and society. They were acutely aware of their poor health and the prospect of mortality linked to drug use. If drug users failed to escape from addiction, taking optimal care of their own health was not deemed a high priority. As a result, even PWID who had an adequate knowledge of HIV might share drugs, needles, and syringes.

*Who will care about me*? *People do not even talk to us properly*. *Now*, *my family members restrict even my home entrance*. *I was nobody in the family and the society*. *So what will they expect from me*? *(key informant interview with a PWID*, *Unemployed)*

Within this context of low esteem, the highest priority was to avoid heroin withdrawal symptoms.

PWID are at considerable risk of HIV and HCV transmission in Bangladesh, as documented in a 2011 national serological survey [1]. A high rate of NSS behaviour is documented in several other studies [1–4]. Despite possible underreporting of NSS, we were able to characterize NSS as a behaviour embedded within the social and cultural life of PWID in our study population of Rajshahi PWID. We found, as did other studies, that conscious collegial reciprocity is one of the underpinnings of NSS. Social organization of PWID bound them together through fictive kinship frameworks with family-like dynamics. The organizational benefits of reciprocal relationship led to drug sharing and NSS. This is not a new observation; NSS and reciprocity as a conscious act among PWID was described in the USA in the 1980s [5, 6]. We found that reciprocal relationships are a strategic behaviour that helps to meet drug needs during times of crisis, mitigating drug access when PWID do not have resources to buy heroin and enhancing sustained peer relationships.

Maintaining a NSS group was also an important survival strategy to continue income flow either from illegal or legal work. This was not often represented in other studies that more typically characterized PWID as passive. Marginalization did not mean voicelessness in Rajshahi. PWID reported resisting misdeeds against them using their groups as defenders. However, addicts, even supported by their NSS group, have more difficulty producing meaningful resistance to politico-administrative personnel that posing threat to carrying needles/syringes, given popular, political, and law inforcement views of drug addiction as immoral and illegal.

Heightened drug effects were also cited as a motive for NSS, but was likely more psychological than physiological. Group conversations highlighted their views that NSS activities were a sort of “high” in and of themselves. This kind of belief indicates the challenges facing risk reduction programs that must educate PWID about the benefits of sterile needle/syringe use, even as users highlight what they like about NSS.

We found evidence to support the findings of other investigators that poor economic condition predispose for NSS [7, 14–16]. Maintaining an equitable distribution of drugs among the cost sharing PWID incentivizes NSS risk behaviours. Our study participants maintained equitable drug distribution by pushing drug volumes from the same syringe as per one’s financial contribution to the drug purchase.

PWID reported obstacles to getting new needles/syringes from harm reduction programmes. Like Chakrapani et al publishing in 2011 and Magura et al in 1989, found that PWID risk harassment when they carry needles/syringes whether for imminent use or needle exchange [8, 16]. A lack of safe storage places, inconvenient time-schedules for needle-syringe distribution, unequal power relations between sometimes poorly trained field workers and PWID, and biased and judgmental attitudes of field workers were cited as important issues. Many misconceptions were identified here, as in other studies. For example, both we and Azim et al found that NSS with a family member or someone who appeared healthy was viewed by PWID as to not carry health risks [28]

We agree with Malow et al that anxiety and depression can augment drug-using risk behaviours [20]. Our qualitative data suggests that low self-esteem contributed to NSS episodes. Low self-esteem is embedded with stigmatization, marginalization, maltreatment, and poor physical conditioning. Withdrawal symptoms supersede concern for health, as reported by others [7, 16]

Finally, abundant evidence confirms that needle exchange programmes have reduced HIV transmission rates among PWID globally [15]. An HIV intervention functional framework that is compatible with what PWID told us in this study would be ideal has been described as follows:

*“The intervention consists of delivery of sterile needle-syringe at free of cost either from drop-in-center or from vending points*. *… As well as providing clean needles*, *a needle exchange scheme can also act as a gateway through which users learn about safe injection practices and equipment disposal*, *safer sex education*, *access to other prevention services such as substitution therapy*, *and referral to treatment*.*”* (http://www.avert.org/needle-change.htm)

However, we argue that only a steady and convenient supply of sterile needles/syringes and safer sex/HIV education can begin to halt transmission of STI, HIV, HCV, and other blood-borne infections. Bangladesh risks an expansion of HIV among PWID at the same magnitude as experienced in Pakistan since 2002 [33]. Risk behaviours are linked to multiple and complex social and cultural conditions. NSS behaviours are not isolated or independent phenomena; rather the basis of NSS is dependent on multiple interconnected factors. It is also possible that the exact motivations for NSS may be only partially generalizable, with some features that are specific to particular PWID subculture and context. Interventions must take into account these diverse sociocultural contexts to scale up quality risk reduction programmes. In particular, advocacy must extended to addicts family, neighbor, community leader, and pharmaceutical shop personnel to reduce marginalization and to improve free access to needles/syringes. More work with relevant security personnel is needed, such that police become allies in the risk reduction efforts. Field workers must also receive training and supervision to treat their clients with dignity and respect. Sessions for behavior change communication (BCC)/information, education, and communication (IEC) may address NSS values, such as collegial reciprocity, friendship and risk sharing, and access to needles/syringes that promulgate NSS. Peer concepts such as the ‘old users cum leader’ may help catalyze behaviour change. We think that misconceptions are so substantial that workers may need to address HIV and HCV-related misconceptions even after several awareness session have been held.

Our findings might be more comprehensive if we able to identify what makes a few not go for NSS and take into account female injection drug users and their dynamics of NSS. Further research on the issues might enrich overall understanding of NSS behavour. Why misconception to risk of HIV and HC and others prevailed might not get due attention. However, what we present so far generate from adequate cross check to their fluctuated opinions, which being necessary while interviewing with PWID.

## Conclusion

NSS behaviours are entwined within the social organizations of Bangladeshi PWID. Poorly functioning harm reduction programmes that have needle/syringe exchange at inconvenient times and promulgated by unsympathetic staff contribute to NSS. Interventions must take into account these diverse socio-cultural and programmatic contexts to scale up high quality, effective risk reduction programmes.
